# Artificial Compound Eye Systems and Their Application: A Review

**DOI:** 10.3390/mi12070847

**Published:** 2021-07-20

**Authors:** Huu Lam Phan, Jungho Yi, Joonsung Bae, Hyoungho Ko, Sangmin Lee, Dongil Cho, Jong-Mo Seo, Kyo-in Koo

**Affiliations:** 1 Department of Electrical, Electronic, and Computer Engineering, University of Ulsan, Ulsan 44610, Korea; phlam.ulsan@gmail.com; 2 Department of Electrical and Computer Engineering, Seoul National University, Seoul 08826, Korea; myrmidon91@snu.ac.kr (J.Y.); dicho@snu.ac.kr (D.C.); callme@snu.ac.kr (J.-M.S.); 3 Department of Electrical and Electronics Engineering, Kangwon National University, Chuncheon 24341, Korea; baej@kangwon.ac.kr; 4 Department of Electronics Engineering, Chungnam National University, Daejeon 34134, Korea; hhko@cnu.ac.kr; 5 Department of Biomedical Engineering, Kyung Hee University, Yongin 17104, Korea; sangmlee@khu.ac.kr

**Keywords:** artificial compound eye, microlens array, metalens, high resolution imaging, large field of view imaging, curved image sensor, biomimetic system

## Abstract

The natural compound eye system has many outstanding properties, such as a more compact size, wider-angle view, better capacity to detect moving objects, and higher sensitivity to light intensity, compared to that of a single-aperture vision system. Thanks to the development of micro- and nano-fabrication techniques, many artificial compound eye imaging systems have been studied and fabricated to inherit fascinating optical features of the natural compound eye. This paper provides a review of artificial compound eye imaging systems. This review begins by introducing the principle of the natural compound eye, and then, the analysis of two types of artificial compound eye systems. We equally present the applications of the artificial compound eye imaging systems. Finally, we suggest our outlooks about the artificial compound eye imaging system.

## 1. Introduction

The design of devices mimicking the structure of natural organisms and their functions or behavior is known as biomimetic [1]. Nowadays, most of the biomimetic vision system is inspired by vertebrate and insect eyes due to their outstanding optical system [2,3]. The optical imaging systems mimicking principles of the vertebrate have been extensively and ubiquitously used in many applications, such as mobile phone cameras and digital cameras, because they could achieve a high spatial resolution [4]. However, this type of imaging system suffers from the restriction of miniaturization and narrow field of view (<80°) [5,6]. Despite the rapid progress in electronic manufacturing technology, the size of current micro-cameras is rarely smaller than 1 × 1 × 1 mm^3^ [7] because physical and technological limitations prevent further size downscaling. Further downscaling of a single pixel size is less than the diffraction limit (approximately 0.25 μm), which will reduce the spatial resolution and increase noise drastically.

A large field of view (FOV) has been implemented by fisheye lens, catadioptric lens, and rotating camera [8,9,10]. The fisheye lens suffers from heavy distortion occurring at its edge [11]. This distortion hinders the fisheye lens from being used for high-quality image acquisition. Furthermore, its resolution is not uniform throughout, owing to the off-axis aberrations. In case of the catadioptric lens and the rotating camera, they can only provide a large FOV for a static scene due to their mechanical rotating structure [11].

To overcome the above-mentioned limitations of the imaging system, researchers are now considering eyes in nature. In comparison with vertebrate’s single-aperture eye, insect’s compound eye is characterized as large FOV, low aberration, low distortion, high temporal resolution, and infinite depth of field [12,13]. Diverse variants of artificial compound eye have been proposed and studied.

In 2015, Seo et al. categorized artificial compound imaging system into three types: Microlens, micromirror, and gradient refractive index (GRIN) lens array type in view of optic components [2]. Since the Seo’s review, no report on the micromirror and the GRIN lens array has been made by researchers. As a consequence, many researchers are now intensively studying metasurface lens. Based on this trend, this paper studies the metasurface lens and the microlens array, but excludes the micromirror lens and the GRIN lens array. The remaining part of this paper is organized as following: Section 2 introduces the principle of the natural compound eye. Section 3 presents the artificial compound eye using microlens array. Section 4 illustrates the artificial compound eye using metasurface lens. In Section 5, we discuss applications of the artificial compound eye, and then Section 6 is the conclusion and outlook on the artificial compound eye.

## 2. Natural Compound Eye

Natural compound eyes have been subject to scientific research for more than a century. In this section, we cover only the basics of natural compound eye vision.

In nature, eyes can be divided into two major groups: Single aperture and compound eyes [14]. The advantage of single-aperture eyes is high sensitivity and resolution. Unlike single-aperture eyes, compound eyes have a poor image resolution however, they possess a large view angle and the ability to detect fast movement due to their faceted structure [15]. A natural compound eye has ten to tens of thousands of ommatidium spherically arranged on the surface of the eye depending on the insect species, most of which are hexagonal in shape with diameters ranging from 10 um to 140 um [16]. Each ommatidium can be considered an individual imaging system that typically encompasses a microlens, crystalline cone, and rhabdom. Every single ommatidium can collect incident light and reproduce partial images of the objective separately, and the entire image will be constructed by these imaging mosaics, making the FOV of the compound eyes exceeds 180. Considering the light pathway, the compound eyes of insects are generally categorized into two types of structure: Apposition and superposition compound eyes [15,17,18,19]. For the apposition compound eye, each lenslet receives light from a single channel and there is no optical crosstalk between two neighboring channels, whereas in the superposition compound eye, each receptor receives light from several neighboring channels. Therefore, the superposition compound eye is much more light-sensitive, making it common in deep water creatures living in dim light. However, the main limitation of superposition eyes is the aberrations, which are the consequence of a light combination from different ommatidia.

### 2.1. Apposition Compound Eye

**Figure 1:**
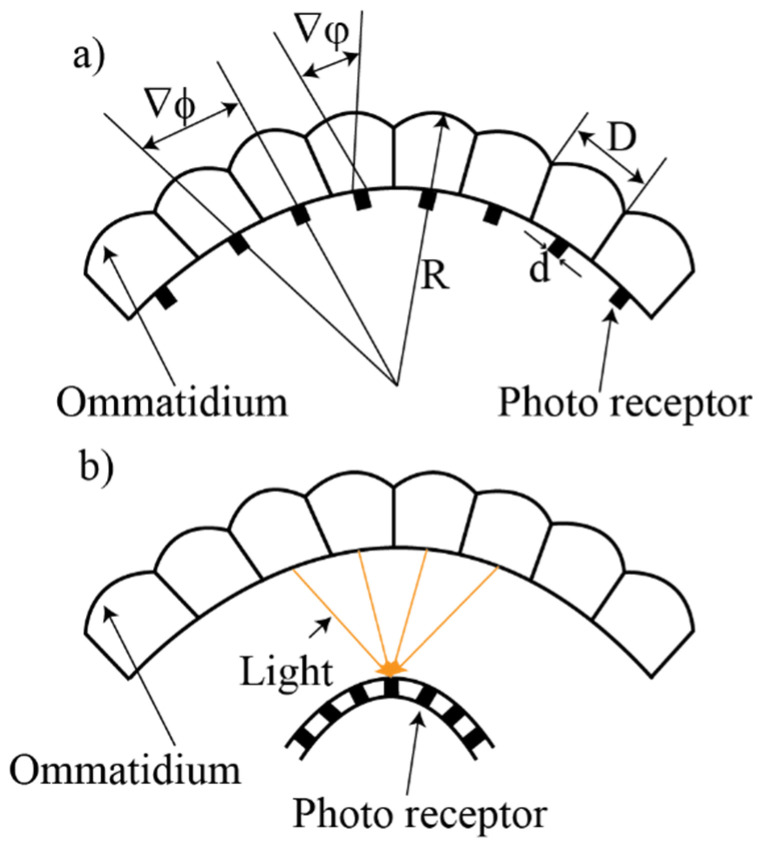
The types of the natural compound eye: (**a**) The apposition compound eye and (**b**) superposition compound eye.

Apposition compound eyes are commonly found in day-active insects such as wasps, fly, dragonfly, bees, and cockroaches [20,21]. The natural apposition compound eyes encompass numerous identically and repeated visual units, the ommatidium, which is formed from single microlens-receptor units as shown in Figure 1a. The light through each ommatidium is received by only one photoreceptor [14]. To prevent the optical crosstalk between the adjacent ommatidia, the pigment cell is added to their intermediate space [22].

Furthermore, the apposition eyes have small lenses (10-µm diameter) resulting in unreliable visual signals at low light intensity. Therefore, they work best at highlight intensities. The final image is composed of the responses of independent ommatidia. The information from ommatidia is integrated into the brain to obtain a final image.

However, a small lens size is the main drawback of the apposition eye. It diffracts the light larger such that only small light reaches the rhabdoms. In some flies, the rhabdom is divided into multiple light guides to improve their light sensitivity. The signals from the individual light guides are summed neutrally to produce a stronger signal for the brain. This type of eye is often referred to as apposition-neural superposition eyes.

### 2.2. Superposition Compound Eye

Light from multiple facets is guided to a common receptor to form a single erect image of the object [14]. This shows that the superposition compound eyes are much light sensitive than the apposition eyes. Moreover, the imaging resolution of the superposition compound eye is about ten times higher than the apposition compound eye [23]. In superposition eyes, the visual units do not work independently because up to hundreds of adjacent facets support each other in capturing light, thereby enhancing the aperture when compared to the apposition eyes. This type has primarily evolved in nocturnal insects and deep-water crustaceans that live under low light conditions [19].

**Figure 2:**
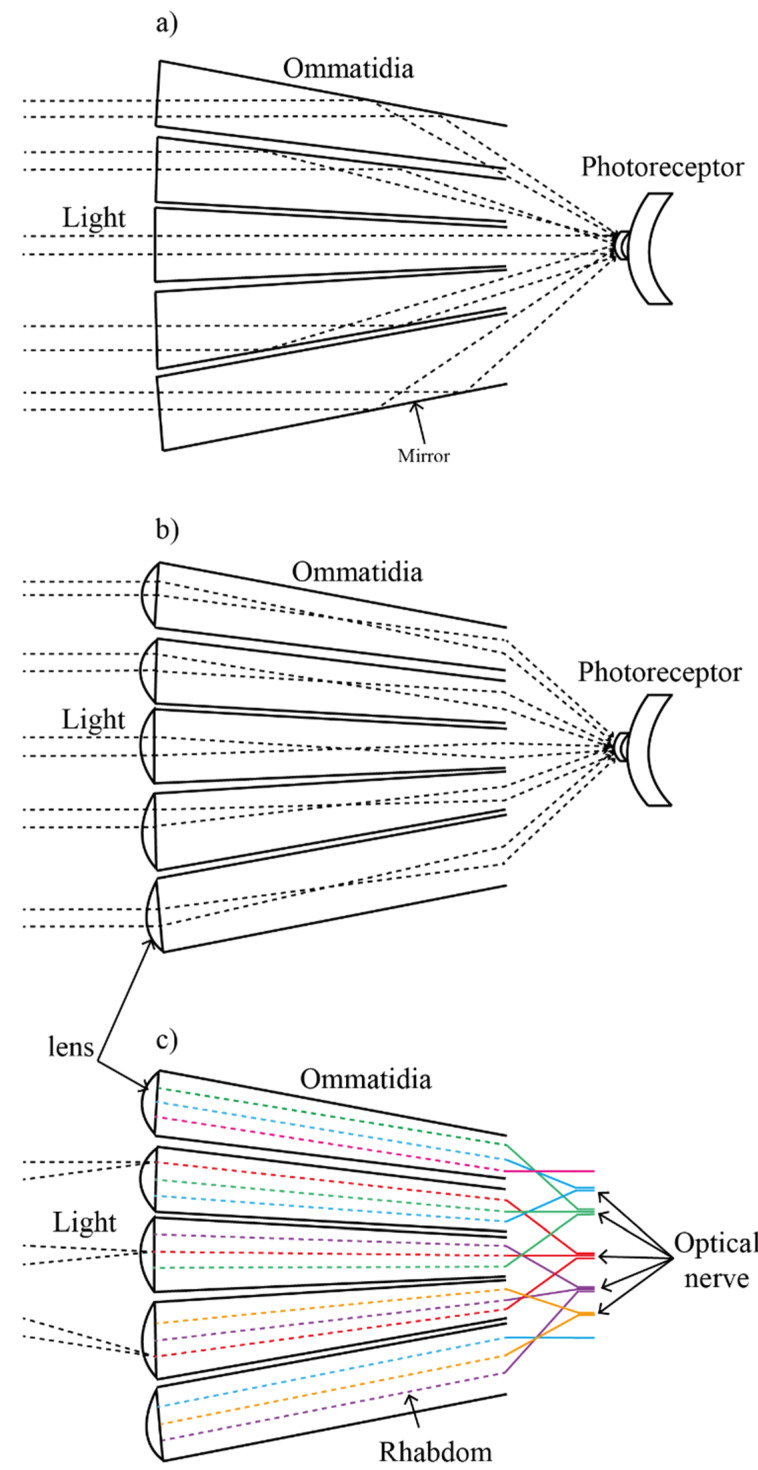
Types of the superposition compound eye: (**a**) The reflective supposition compound eyes, (**b**) the refractive superposition compound eyes, and (**c**) the neural superposition compound eyes.

Superposition compound eyes can be subdivided into three types: Reflective, refractive, and neural superposition, as shown in Figure 2. The reflective superposition eyes have long rectangular walls that operate as biological mirrors. They also have a transparent gap like the refractive superposition eyes, but they use corner mirrors instead of lenses to reflect light to the retina. Therefore, the images are formed using only the reflections in the mirrors. This type is used mostly by decapod crustaceans such as shrimp and lobster (Figure 2a). Another variant is the refractive superposition eyes found in nocturnal insects. In refractive superposition eyes, light is guided to the photoreceptor using the refraction of ommatidial lenses (Figure 2b). Moreover, there is a gap between the lens and photoreceptor in sharing photons efficiently from the adjacent ommatidia. The refractive superposition eyes have a magnification of photosensitivity, which is improved three times than that of simple eyes.

For the neural superposition eyes, each ommatidium consists of several rhabdoms which are superimposed on the one and the same optic nerve (Figure 2c). Therefore, the incident light from different lenses is accumulated on different rhabdoms in one ommatidium. Then, one optical nerve composes different rhabdoms together. They can provide a higher resolution without increasing the ommatidium. This type of superposition eyes is found in many dipteran flies such as the housefly. A comparison of different eyes and related parameters is shown in Table 1.

## 3. Artificial Compound Eye Imaging System Using Microlens Arrays

Based on the fabrication technology development of microelectronics and microlens array (MLA), we divided these artificial compound eye imaging systems into two groups: Planar-type and curved-type artificial compound eye.

### 3.1. Planar-Type Artificial Compound Eye

Most compound eye systems have been designed and fabricated on planar substrates, due to commercially available image sensors, such as a charge-coupled device (CCD) or complementary metal oxide semiconductor (CMOS) that are manufactured on planar wafer.

**Figure 3:**
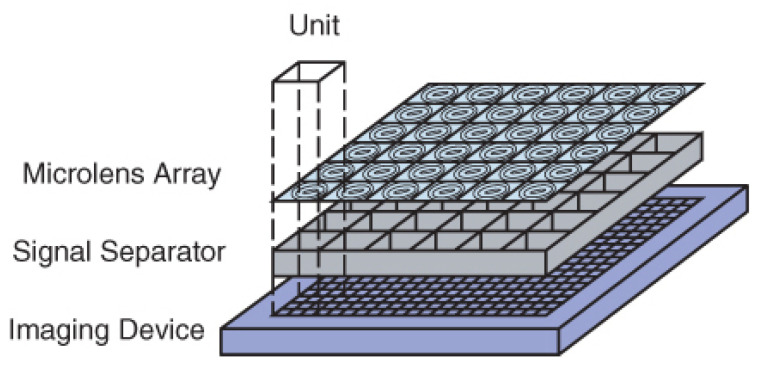
The structure of the TOMBO system. Reprinted with permission from [28] © The Optical Society.

A compact image-capturing system called a thin observation module by bound optics (TOMBO), presented by Tanida et al., mimics a dragonfly’s apposition compound eye [26]. The system consists of three components: An MLA, a separation layer, and a photodetector array. Figure 3 shows the structure of TOMBO systems. Here, a microlens and a photodetector, including multiple photosensitive cells form an individual imaging system (unit). The separation layer is utilized to prevent signal crosstalk among units. Owing to the small size of the elements, the structure of TOMBO is simple, but high productivity is expected. In the first version of the TOMBO system, a conventional CCD chip is used for the photodetector array combining a planar MLA [26]. However, they did not demonstrate the compactness of the TOMBO architecture through this version. To demonstrate the effectiveness of the TOMBO architecture, the author has constructed the prototype system with a refractive microlens array and a CMOS for photodetector array [27]. They showed that the system is simpler and thinner than the traditional imaging system. Specifically, the TOMBO system prototype has a 650-µm focal length and 250-µm diameter of the MLA (16 × 12). The pixel pitch of the CMOS image sensor is 12.5 µm and the number of pixels per unit is 20 × 20, respectively. However, the captured compound image only has 320 × 240 pixels with 8-bit depth, which is the limitation of this system. The color imaging is one indispensable requirement for current imaging devices. To overcome the shortcoming, two implementation methods are presented in the TOMBO system [28].

One is color separation by pixels, and the other is color separation by units. In color separation by pixels, the basic color (red, green, and blue) channels are assigned to the single pixels. It has its applicability in the commercial color CCD for the imaging device. Unlike color separation by pixels, color separation by units assigns color channels to the individual units. Thus, it is an easy fabrication of color filters attached to the imaging device, and it has a relatively relaxed imaging condition. Despite there being a miniaturization artificial compound eye system provided, the FOV of this system was about 35°, which is small [29] because of the limitations of the planar substrate.

Additionally, another planar artificial compound eye with planar MLA called the Apposition Compound eye objective (APCO) was proposed by Duparre et al. [30].

**Figure 4:**
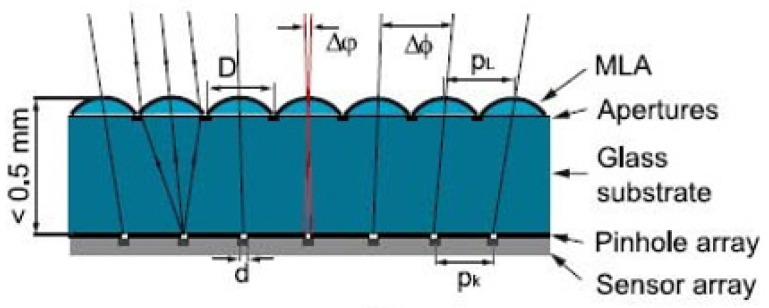
The schematic diagram of the APCO system. Adapted with permission from [33] © The Optical Society.

The APCO is constructed with a microlens and a pinhole array in its focal plane, as depicted in Figure 4. The master structures for the MLA are fabricated by lithographic patterning of photoresist and a subsequent reflow process. The task of the pinhole array is to cover a detector array placed behind the substrate with larger pixels to achieve the desire resolution. The pitch of the pinholes differs from the lens array pitch to enable an individual viewing angle for each channel. Thus, behind each microlens, a small sub-image of the object is generated. Generally, the parameters of the imaging system, such as the overall thickness, F-number, and diagonal FOV are 320 µm, 2.6, and 21°, respectively. The FOV can be extended to 42° by adding a Fresnel lens. The pinhole diameter has a range from 2 µm to 8 µm, while the resolution of the CMOS image sensor is 128 × 128 with a 69-µm pixel pitch and the angle resolution of the APCO system is 1.5 LP/deg (Line pair per degree). To prevent the ghost images that result from the crosstalk effect, an opaque wall was placed between the adjacent optical channels [31]. Although the artificial compound eye systems were miniaturized, they had a small FOV. Furthermore, to enlarge the FOV of the artificial compound eye imaging system, Duparre et al. proposed an artificial compound eye imaging system called a cluster eye (Cley) [32], which mimicked the neural superposition compound eye.

**Figure 5:**
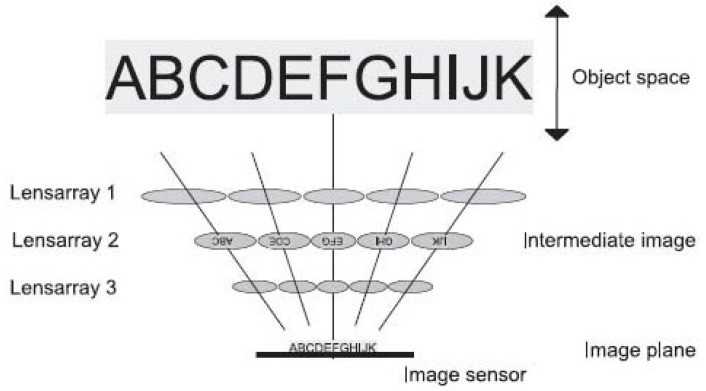
The working principle of the Cley system. Reproduced with permission from [32] © The Optical Society.

The Cley system includes three MLAs with different pitches, consisting of a field lens and a field aperture array. The structure of the Cley system is described in Figure 5. The MLA with different pitch can be viewed as a cluster of single pupil’s micro cameras, which have tilted optical axes to obtain the overall FOV. The lens shapes are fabricated by the reflow of photoresist cylinders on ellipsoidal bases. The lenses are subsequently transferred into fused silica by reactive ion etching. Arrays of apertures with focusing, field lens, and relay lens array are applied onto the corresponding lens arrays by chromium etching or lift off. Each channel image has only a small viewing angle. The amount of overlap of the sub-image is dependent by the aperture size of the filed stop array. A Cley imaging system has 21 × 3 channels, 70° × 10° FOV, and 4.5 mm × 4.5 mm. Each channel has an individual FOV of 4.1° × 4.1°. The size of the individual micro-images is 192 µm × 192 µm. The system length of the Cley system is only 1.99 mm. For the channel, a resolution of 3.3 LP/° is obtained. Compared with APCO, the image resolution has been greatly improved. However, the structure of the system is quite complicated in fabrication and the thickness is nearly ten times that of APCO. Moreover, this structure is affected by astigmatism in a large incident angle. Therefore, each ommatidium needs to be designed carefully to correct the astigmatism. To solve this problem, we proposed using chirped arrays for ellipsoidal microlenses. Each microlens is designed carefully to correct the astigmatism. The homogeneity of the imaging resolution has been significantly enhanced since each optical channel is precisely corrected for its responsible FOV [34].

Nevertheless, the disadvantage of the type of artificial apposition compound eyes system is the inverse relationship between the resolution and sensitivity [31]. Increasing the diameter of the pinhole to improve the sensitivity results to a decrease in the resolution. To solve this problem, an imaging system that relies on the principle of a neural superposition compound eye was proposed by Bruckner and Duparre et al. [33]. The fabrication techniques for the artificial neural superposition eyes system are very similar to the APCO system. The difference between the two systems is that the fabrication technique exhibits multiple pixels in each channel. The effect number of channels is 52 × 43. The diameter and the F-number of the microlens are 125 µm and 2.5, respectively. The pinhole diameter is 3 µm and the FOV of this system is up to 23° × 21°. The optical thickness is 450 µm. Based on the result of the measurements of the signal to noise ratio, the ability of the artificial neural superposition eye to increase sensitivity and to suppress noise is demonstrated. The signal noise ratio is three times larger than in the conventional system because each pixel of the final image results from an average of nine pixels observing the same point in object space. Using redundant sampling with multiple pixels per channel allows it to break the inverse relationship of the resolution and sensitivity of artificial apposition compound eyes.

**Figure 6:**
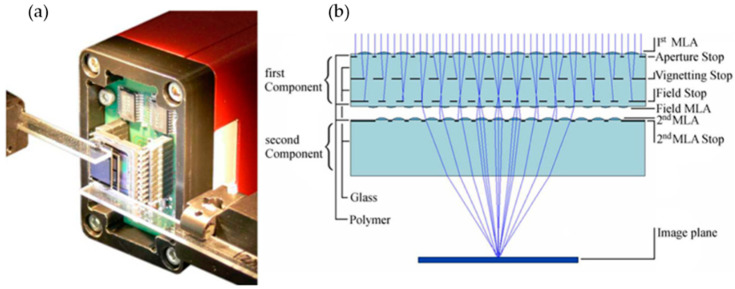
(**a**) The fabricated Gabor superlens. (**b**) The optical principle of the Gabor superlens. Reprinted with permission from [23] © The Optical Society.

Furthermore, another well-known natural superposition compound eye imaging system “Gabor superlens” is proposed by Stollberg et al. [23]. The structure of this system consists of two MLAs, which are separated by the sum of their focal lengths (Figure 6). In the system, the dimension of the first MLA pitch is larger than the second to form a real and erect image of the object. In another way, the imaging system results in a virtual image. The authors utilized photolithography, a reflow progress, structure transfer, fabrication of the molding tool, and ultraviolet molding to manufacture the Gabor superlens. The number of channels is 15 × 15. The effective focal length, the effective entrance pupil diameter, and the F-number of the Gabor superlens are about 2.4 mm, 864 µm, and 2.8, respectively. The spatial and angular resolutions are 49 LP mm^−1^ and 3.0 LP/°. The full horizontal FOV is 29°, and the effective number of image points is 156 × 156. The dimension size of the artificial compound eye imaging system is 2.8 mm × 2.8 mm. Compared to the Cley imaging system, the Gabor superlens with two MLAs has relatively low resolution.

Later, inspired by the compound eye of a parasite of wasps called Xenos Peckii, Druart et al. introduced the idea to read out the complete partial image within each imaging channel. The partial images are then stitched together to form a total image with high resolution through image processing [35]. Then, Bruckner et al. followed this idea and reported an artificial compound eye imaging system called the Electronic Cluster Eye (eCley) [36,37,38]. The eCley has an overall thickness of only 1.4 mm, the number of channels is 17 × 13, and the diameter and F-number are 0.38 mm and 3.7, respectively. Although the system has a small dimension, it can obtain a large FOV (58° × 64°) with a VGA resolution (700 × 550). To reach a VGA resolution, a customized image sensor in a large active area is needed. Thus, this is the main drawback of the eCley system. To solve this problem, Meyer et al. introduced an artificial compound eye imaging system with optical stitching of segments called the oCley system (Optical Cluster Eye) [39].

**Figure 7:**
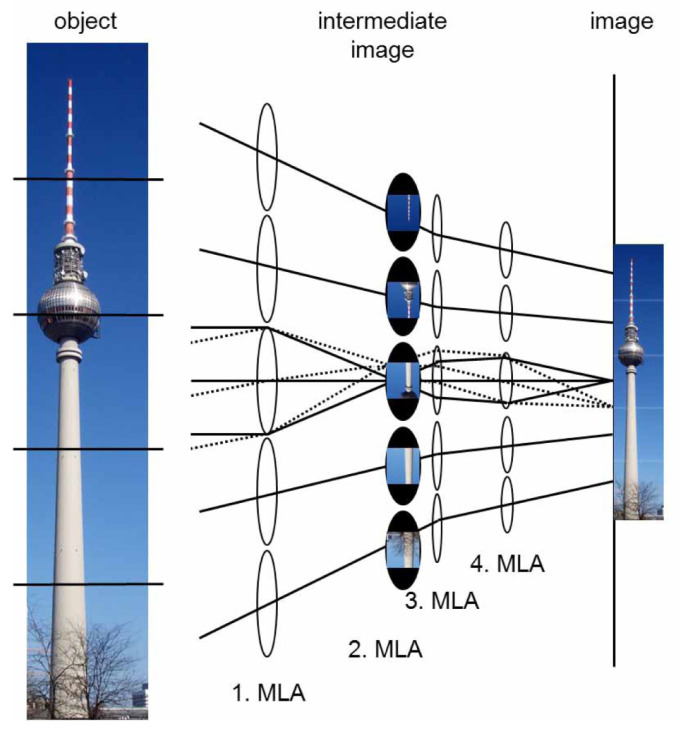
The working principle of the oCley system. Adapted with permission from [39] © The Optical Society.

The oCley is an imaging system based on a multi aperture architecture. The system includes four MLAs with different pitches, as shown in Figure 7. The first MLA concentrates on the rays into the intermediate image plane. Then, the intermediate images are transferred by the third and fourth MLA onto the image sensor plane. The second MLA operates as field lens array, which minimizes vignetting and decreases the lens diameters at subsequent arrays. The optical axes of the different channels are tilted to each other to transmit a wide range of view. Compared with the prior system, the system has an extra array that is located between the field lens array and the last lens array to tilt the optical axes. The tilt leads to an increase in irradiance at the image plane and a decreased crosstalk between adjacent pixels. Moreover, there is no image processing related to the sub-image stitching; a real erect image is therefore created by a conventional image sensor. The oCley system can capture images at a VGA video resolution (640 × 480). The FOV of this system is up to 53.2° × 39.9°. The spatial resolution and angular resolution are 155 LP/mm and 4.2 LP/°, respectively. The F-number and the effective focal length are 6.7 and 1.39 mm, respectively. The image size is 1.44 mm × 1.08 mm, the thickness of the system is 1.86 mm, and the pixel size is 2.25 µm × 2.25 µm.

**Figure 8:**
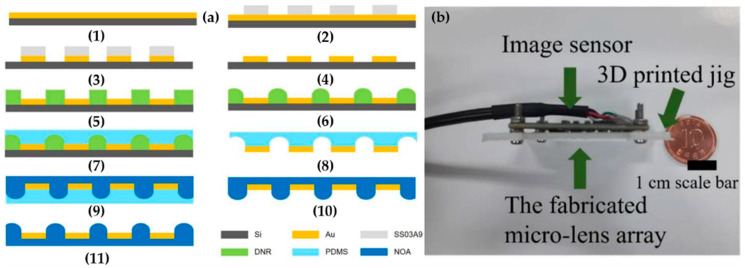
(**a**) The fabrication process of the microlens using the soft lithography process. (**b**) The structure of artificial compound eye system. Reproduced from [5]. CC by 4.0.

Although there is an improvement for the FOV and the image resolution in the above artificial compound eye systems, it is not significant when compared with the commercial camera. Recently, Seo et al. proposed a multi-aperture imaging system that has a larger range of view than a 35-mm conventional camera with a compact lens system. The imaging system consists of a MLA, a lens adjusting jig, and a CCD image sensor. In this system, there is a light screening layer patterned among all the individual microlens to prevent light interference. The soft lithography process is used in integrating the layer with the individual microlens (Figure 8). The lens adjusting jig is utilized to calibrate the distance between the MLA and CCD sensor to focus every microlens’ image. The system can reach a resolution (1920 × 1080) and provide a FOV of 100° [5]. However, the dimension of the system is not significantly reduced due to the size limitation of the commercial image sensor.

**Figure 9:**
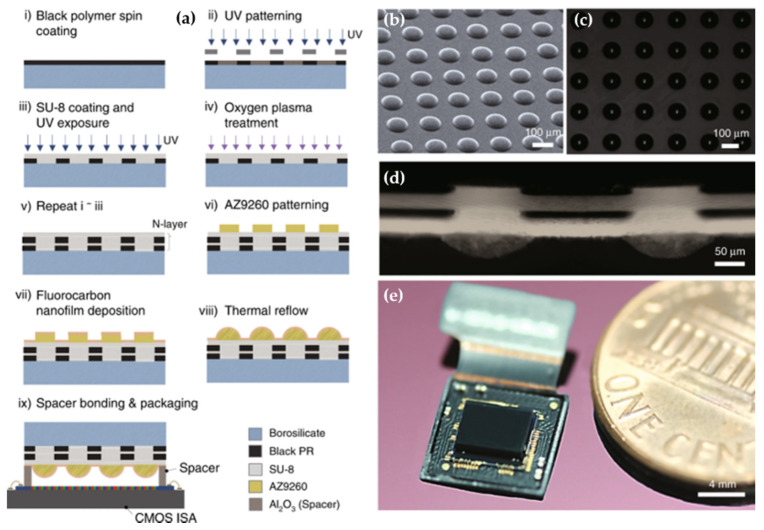
(**a**) The micro fabrication method of the micro-optical elements. (**b**) The scanning electron microscopy of MLAs. (**c**) The optical microscopy images of MLAs. (**d**) The cross-sectional optical image of the Micro Optical Element. (**e**) The structure of a fully packaged ultrathin arrayed camera. Reproduced from [40]. CC by 4.0.

Recently, an ultrathin arrayed camera inspired by insect eye structure for a high resolution is proposed by Kim et al. [40]. Figure 9a presents the fabrication technology of the micro-optical elements that have been used. The scanning electron microscopy, the optical microscopy images, and the cross-sectional optical image of the microfabricated MLA are described in Figure 9b, Figure 9c and Figure 9d, respectively. The system includes an inverted MLA, multilayered pinhole arrays, and gap spacers on an CMOS image sensor, as shown in Figure 9e. The multilayered pinhole allows high-contrast imaging by eliminating the optical crosstalk among microlenses. The fully packaged system shows a total track length of 740 µm and a FOV of 73°. The comparision of the performance of the planar-type artificial compound eye imaging systems is shown in Table 2.

### 3.2. Curved-Type Artificial Compound Eye

#### 3.2.1. Curved Microlens Array Integrated to a Planar Image Sensor

Artificial compound eyes with a planar structure are easy to design and fabricate, but the range of view is exceedingly small. To address this problem, most natural compound eyes have curved structures. In this section, we review the artificial compound eye with a curved MLA and planar image sensor.

A laser-based fabrication process is a popular method in manufacturing curved-type artificial compound eyes. Liu et al. manufactured an inspired artificial compound eye structure. This system contains many close-packed microlenses onto curvilinear surfaces (7600 hexagonal-shaped microlenses) [41].

**Figure 10:**
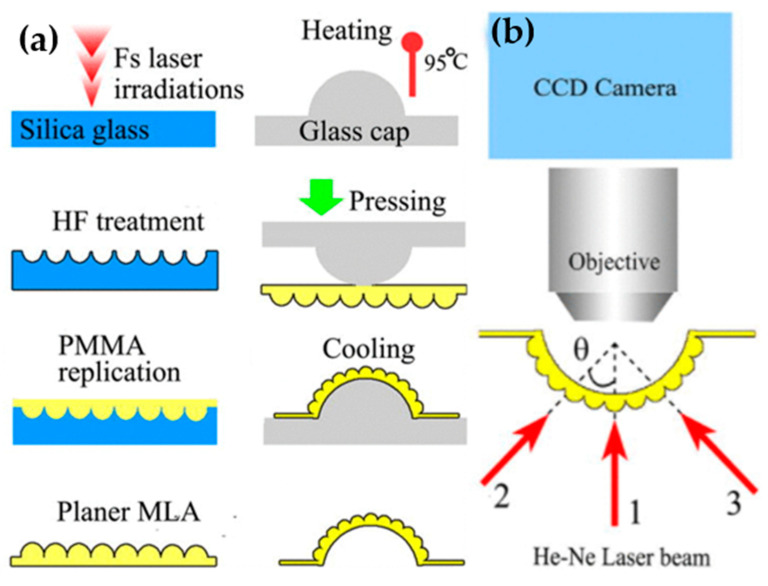
(**a**) The schematic diagrams of the fabrication process. (**b**) The optical setup experiments. Reproduced from [41], with permission of AIP Publishing.

**Figure 11:**
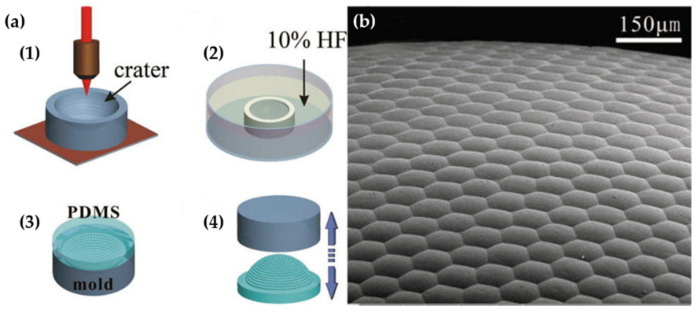
(**a**) The fabrication process of curved MLA using the femtosecond laser enhanced chemical wet etching technique. (**b**) The SEM picture of the fabricated curved MLA. Reprinted from [43], with permission of AIP Publishing.

The fabrication of microlens was implemented in two steps, as depicted in Figure 10. First, the fabrication process started with the manufacturing of planar MLA on the poly methyl methacrylate (PMMA) films using the femtosecond-laser microfabrication and replication process. Second, the concave microlens was fabricated on a silica glass chip (30 × 30 × 2 mm^3^) by transforming the planar PMMA film with MLA to the hemispherical shape (3.5-mm diameter, 1.6-mm dome height) using a previously developed technique in [42]. Figure 10b shows the optical set-up experiment. The focal length, diameter, and height of the fabricated microlens are 92.4 µm, 50 µm, and 7.2 µm, respectively. The interommatidial angle of the curved MLA is about 2°. The theoretical FOV value can reach up to 162°. However, this method needs high precision in the bending process to avoid the distortion of the microlens. To eliminate the distortion, Bian et al. also proposed a method that directly fabricate a concave MLA on curved surfaces based on a femtosecond laser enhancing the chemical wet etching process. Moreover, the fabrication process of the curved microlens is divided into two main steps. Specifically, the femtosecond laser (800 nm, 50 fs, and 1 kHz) is focused on the center of the concave surface (a spherical plano-concave lens with a diameter of 6 mm) of the lens, which is fixed on a 3D stage with only a x-y-z linear stage. Next, using the method of 0.5 s of exposure controlled by a mechanical shutter, a hexagonal-packed array is fabricated without rotation of the sample due to the adjustment the z-axis position before each laser exposure. Then, the lens is immersed in the HF (10%) ultrasound bath for 80 min. Finally, the compound eye-inspired curved MLA is formed by the de-molding process after pouring the liquid polymer into the concave lens followed by a peeling process (Figure 11). Therefore, the artificial compound eye with 3000 positive microlenses of 95-µm diameter close-packed on a 5-mm polymer hemisphere is fabricated [43].

Besides the laser-based fabrication processes to produce artificial compound eyes, other approaches have also been introduced. In fact, Huang et al. present an approach of combining the maskless projection lithography technique with the thermal reflow process to successfully fabricate the biomimetic compound eye (BCE) on a curved substrate [44]. The fabrication process includes a two-step reflow method combining the lithography process. First, a triple-layer photoresist film (top layer, inter-layer, and bottom layer are a positive photoresist from Clariant AZ4620, Shipley S1813, and Clariant AZ4620, respectively) is sticked onto a silicon substrate. Then, the hierarchic microstructures with small cylinders on the big cylinders was obtained through DMD maskless lithography. After that, the spherical microlens of the BCE is formed via the first thermal reflow process with direct contact heating (125° for AZ4620 layer and 170° for S1813 layer). Finally, the second thermal reflow is implemented to establish ommatidia on the spherical microlens, where the sample was upside-down with a 1-mm gap from the hotplate using the glass slides as the blocker. Here, the upside-down reflow strategy is a different point between this system and Wang’s system [45] This method is flexible because of its easily changing structural parameters in both the curved substrate and curved MLA, which is achieved by adjusting the parameters of the hierarchic microstructure.

**Figure 12:**
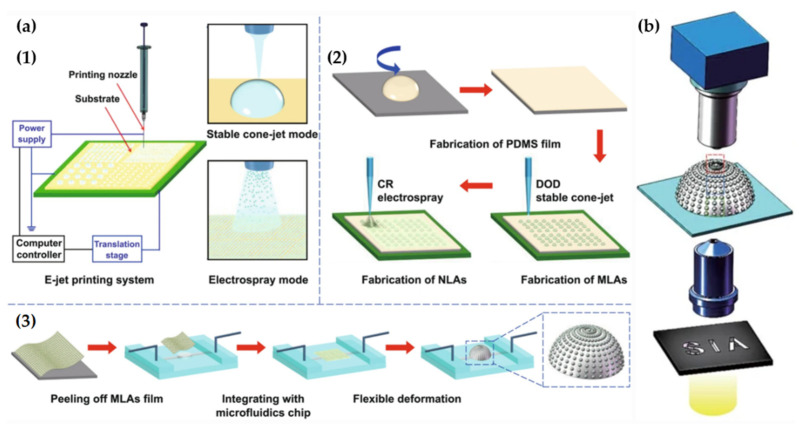
(**a**) The fabrication process of curved MLA using E-Jet printing. (**b**) The structure of curved artificial compound eyes. Adapted from [46]. CC by 4.0.

Furthermore, Zhou et al. presented another approach to manufacture the curved artificial compound eyes [46]. The method uses an electrohydrodynamic jet (E-jet) print, which is an ultra-high-resolution printing technique (Figure 12a). It has proved to be a trustworthy method for maskless and direct manufacturing on various substrates at a large scale. The fabrication process has three steps: (1) Fabrication of the PDMS film; (2) fabrication of hierarchical MLAs and nanolens arrays (NLAs) on the PDMS film; and (3) tunable deformation of the MLAs film via the integrated microfluidics chip. In detail, a liquid PDMS elastomer (Sylgard 184, Dow Corning, USA) and a curing agent were mixed with a weight ratio of 10:1. Then, the PDMS was spin-coated onto a glass substrate, followed by curing the PDMS at 100 °C for 30 min. After that, the UV-curable adhesive was printed on the PDMS film substrate to fabricate the MLAs and NLAs. Subsequently, these arrays were exposed under 355-nm UV light at 1000 mJ cm^−2^ until the droplets were solidified. Finally, the MLAs film was deformed from a planar surface to a curved surface to create artificial compound eyes. Figure 12b shows the structure of curved artificial compound eye systems. It is easy to see that the system has a variable FOV, which ranged from 0° to 160°.

**Figure 13:**
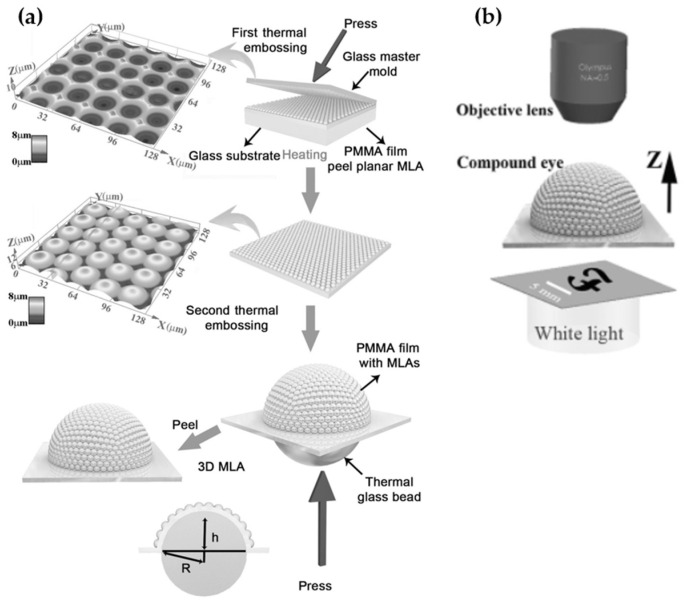
(**a**) The schematic diagram of fabrication process. (**b**) The optical setup experiment of curved MLA. Reproduced from [47] with permission of John Wiley and Sons.

However, the above-mentioned methods hardly control the lenslet geometry and packing due to their intrinsic limitation. These artificial compound eyes usually suffer from a small number of microlenses, which degrade their optical performance. To enhance the number of microlens and imaging performance, Deng et al. proposed artificial compound eyes encompassing 30,000 ommatidia using thermomechanical deformation [47]. The fabrication of microlens was carried out in three steps: Fabrication of the master mold and two thermal embossing steps, as depicted in Figure 13a. First, the master mold was fabricated through a scanned laser irradiation and subsequent hydrofluoric (HF) etching. Then, replication based on thermal embossing was used to obtain a convex MLA. In this case, hundreds of PMMA convex MLAs can be duplicated from a single master mold without forming observable defects. Finally, large-area convex MLAs could be fabricated by thermal embossing. The average diameter and sag height of the convex MLAs were 24.5 and 4.67 µm, respectively. The deformation ratios were 1%, proving that the method can realize convex MLAs with high fidelity. Moreover, the geometry of the microlens can be easily controlled by adjusting laser scanning during the fabrication process. By this method, the artificial compound eyes system is created, which has advanced imaging quality, an exceptionally wide FOV of up to 140°, and low aberration. Figure 13b describes the optical setup experiment.

**Figure 14:**
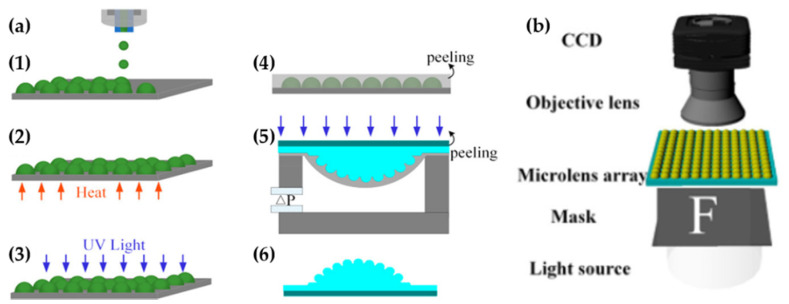
(**a**) The schematic diagram of the fabrication process of an artificial compound eye. (**b**) The schematic setup of the optical system for the measurement. Adapted with permission from [48]. Copyright (2020) American Chemical Society.

In addition, Li and co-worker proposed another approach that can easily control the geometry of a microlens [48]. They used a combination of inkjet printing and air-assisted deformation processes to fabricate the curved artificial compound eye (Figure 14a). Initially, a morphology of planar MLA was manufactured using inkjet printing with a microprinting system (Dimatix Materials Printer (DMP3000), Fuji, Japan). During the process, the geometry and size of microlens were controlled by a number of superposition drops. After determining the required morphology, the planar MLA was obtained using heating and UV-light irradiation. Then, the planar MLA is converse to a curved distribution via membrane deformation. The height of the artificial compound eye was tuned by adjusting the film thickness and negative pressure. Based on the advantages of this method, the artificial compound eye was confirmed with a tunable FOV and tunable acceptance angle. Figure 14b depicts the schematic setup of the optical system for the measurement. The FOV of the ACE reached 140°, while the acceptance angle reached 50°.

Although the above-mentioned methods can easily control the geometry of a microlens, they are not cost-effective, complex, nor do they require a long processing time. To improve these issues, Wang et al. developed a new method for making bionic compound eyes based on the traditional photolithography technology and thermal reflow method [49]. The fabrication begins with a spherical photoresist microlens on the flat substrate, which was fabricated by a traditional photolithographic and thermal reflow method. After the planar photoresist MLA is established, a negative MLA is formed by casting the PMMA solution onto the photoresist microlens arrays at room temperature. At the same time, flat PDMS film was formed by stretching a glass hemisphere mold-coated PDMS. Next, it was pressed onto the PMMA film until it was solidified. As a result, a hemispherical microlens array in PDMS had been successfully obtained after peeling off from the PMMA layer. Finally, a hemispherical compound eye with 6000 microlens was fabricated. The total FOV is about 180° and the numerical aperture (NA) for each ommatidium is about 0.21. Since the developed fabrication process is simple and cost-effective, it is expected to have great potential to be applied in the medica imaging field.

**Figure 15:**
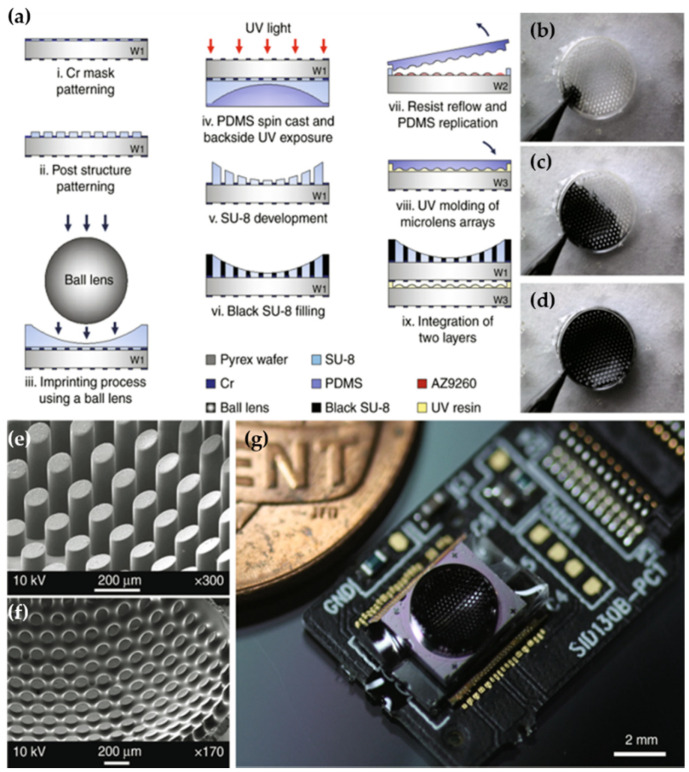
(**a**) The microfabrication steps of the curved MLA. (**b**–**d**) The optical images of 3D capillary filling of black SU-8. (**e**,**f**) The SEM image of concave microprism arrays before and after filling with black polymer. (**g**) The optical image of the fully assembled ultrathin digital camera. Reproduced from [51]. CC by 4.0.

The above-mentioned artificial compound eye imaging systems provide fascinating optical features such as a large FOV and small structure. However, matching the spherical compound eye imaging element and the planar imaging sensor is still a big challenge [50]. To solve this problem, Wang et al. proposed an optical relay system between the curved focal plane of the hemisphere compound eye and the planar focal plane of the CMOS imaging sensor [49]. The optical relay system is designed to convey the focused light into the imaging sensor. It has the same role as the crystalline cone and the wave-guiding rhabdom in natural compound eyes. Additionally, it can offset the distortion of the hemispherical MLA. The focal length of the optical relay system is 5 mm, the maximum range of view is 120°, the relative aperture is about 1/3, and the total optical length is 48.6 mm. Unlike Wang’s system, Keum et al. designed an ultrathin digital camera inspired by the vision principle of Xenos peckii with a concave microprism, a MLA, and apertures on the imaging sensor (Figure 15). Herein, a single microprism tilts the optical axis and guides the focal light to a single microlens. The aperture has a role to block light from adjacent channels. The individual channel is surrounded by a light-absorbing medium to reduce the optical crosstalk between neighboring channels. The system has an FOV of 68° with a diameter of 3.4 mm and a total track length of 1.4 mm [51]. Microlens with a multi-focal length is an approach for matching a curved MLA and planar image sensor, which was proposed by Lian et al. [52]. The MLAs with different focal lengths were first achieved on a polymer and then the planar structure was converted to the curved surface by combining photolithography, hot embossing, soft photolithography, and gas-assisted deformation techniques. The fabricated microlens array consisted of 581 microlens with diameters ranging from 152.8 µm to 240.9 µm. The FOV of the compound eyes exceeded 108°. Based on this method, Liang’s and Han’s groups designed curved artificial compound eyes with different focal lengths microlens to solve the problem of mismatch between the uniform compound eyes and the planar commercially available image sensors. Thus, the system can improve imaging quality of the edge region [53,54]. The parameters and performance of these artificial compound eye imaging system are shown in Table 3.

#### 3.2.2. Curved Microlens Array Integrated to a Curved Image Sensor

The natural compound eyes have the curved MLA that is located on the curved image sensor. To mimic the natural compound eye, an artificial compound eye imaging system should have a curved image sensor. There is a little research on this type of artificial compound eye imaging system due to the limitation in the fabrication technique of the imaging sensor.

**Figure 16:**
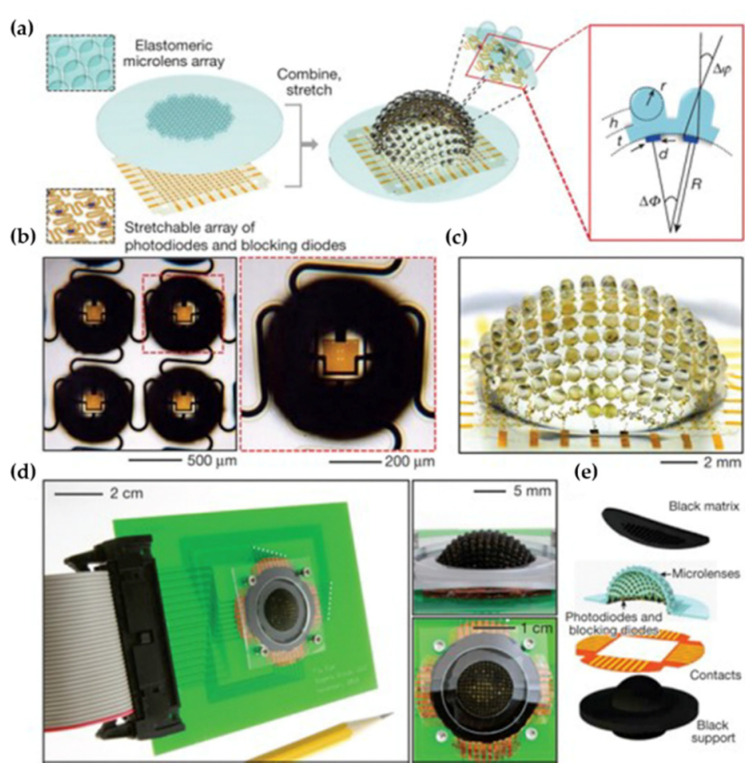
(**a**) The structure of the arthopod-inspired camera. (**b**) The optical micrograph of four adjacent ommatidia. (**c**) The camera system after hemispherical deformation. (**d**) The completed camera mounted on a PCB. (**e**) The view illustration of the components of this system. Reproduced from [55] with permission from Springer Nature Publishing.

An artificial apposition compound eye imaging system mimicking the imaging system of arthropods was developed by Song et al. [55]. The system consists of two main sub-systems. The first sub-system known as the elastomeric compound optical element provides an optical imaging function that includes an array of 16 × 16 PDMS convex microlens with 400-µm curvature radius. The element is formed by casting and curing a prepolymer to PDMS against a precision, micro-machined aluminum mold, and associated fixture. The second sub-system, called thin silicon photodetectors, can realize photodetection and electrical readout. It consists of a matching array of thin silicon photodiodes (160 µm × 160 µm active area) and blocking diodes in an open mesh configuration with the capability for matrix addressing, as shown in Figure 16. The thin silicon photodetectors are manufactured by a sequence of thin-film processing steps conducted on a silicon-on-insulator wafer. To be precise, aligning the bonding of these two subsystems enables each photodiode to be placed at the focal position of a corresponding microlens, which can yield a planar integrated imaging system. The bonded planar layout was transformed into a full hemispherical shape with a radius of 6.96 mm by a hydraulic actuation system. A perforated sheet of black elastomer serving as the screening pigment was placed on the MLA to prevent light crosstalk between the photodiodes. The complete apposition camera system had a FOV of 160° × 160° and a large depth of focus capability due to the very short focal length of each microlens. The acceptance angle and the interommatidial angle of this artificial compound eye imaging system are 9.7° and 11°, respectively.

**Figure 17:**
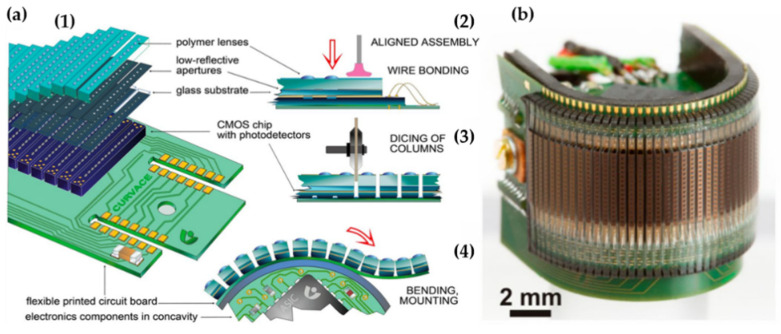
(**a**) The curved design and assembly. (**b**) The picture of the curved prototype. Reprinted from [56] CC by 4.0.

Inspired by the Song’s report, Floreano’s group developed a curved artificial compound eye called CurvACE which includes three composition and functional layers: An optical layer, which is composed of an array of highly transparent polymer microlenses, a photodetector, and an interconnection layer (Figure 17a). The thicknesses of each layer are 550 µm, 300 µm, and 100 µm, respectively. The CurvACE prototype was fabricated by bending a rectangular array of 42 columns of 15 artificial ommatidia down to a curvature radius of 6.4 mm along its longer direction to generate a 180° FOV, as depicted in Figure 17b. The MLA is formed by the reflow of a photoresist on the glass substrate. It was stacked on the photodetector layer, which was constructed in a silicon wafer using CMOS technology. The optical and photodetector layers are aligned at a micrometer accuracy and glued chip wise. Meanwhile, the interconnection layer was formed by a flexible polyimide printed circuit board, which is transferred to the output signals from the individual ommatidium to the processing units. The fabricated CurvACE system had a FOV of 180° × 60°. The diameter of the microlens is 172 µm. The CurvACE system has a compact mechanical design, a volume of 2.2 cm^3^ only, and weighing 1.75g only [56]. The parameters and performance of these artificial compound eye imaging system are illustrated in Table 4.

**Figure 18:**
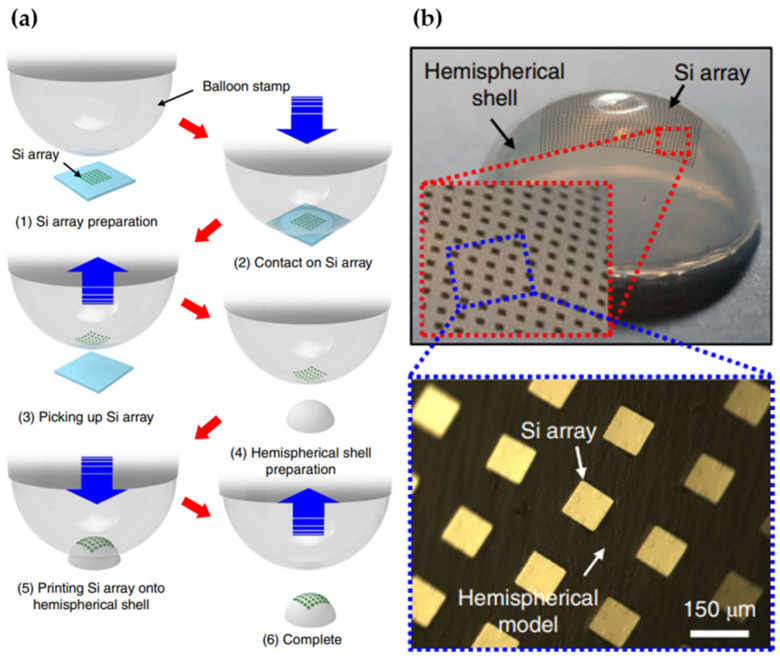
(**a**) The fabrication process of 3D curved optoelectronics using CAS printing. (**b**) The top image of CAS-printed Si array on a hemispherical shell. Reproduced from [60] with permission from Springer Nature Publishing.

Due to the absence of robust manufacturing technologies, creating 3D curved image sensors is incredibly challenging. To overcome the drawback, some innovative methods are proposed for the fabrication of 3D optoelectronics. Liu et al. proposed a deterministic folding method for deforming a 2D polymer sheet into 3D forms using external light. The surface of sheets is integrated with the black ink patterns by a desktop printer, and followed by irradiation of light. The absorbed light of the inked region is converted to heat, which causes the sheet to fold within seconds due to a localized gradient of shrinkage across the thickness of the polymer sheet [57]. It is a simple method for the programmed self-folding of 2D sheets into 3D forms, but the technique is restricted in its ability to access non-developable geometries, such as spherical or arbitrarily complicated curvilinear forms. Other approaches are three-dimensional printing and pad printing, which have been used to transfer electronics inks directly onto a curved surface [58,59]. The authors used the e-jet technique to form complex geometries of electrically conductive patterns on the highly curved substrate (the radius of curvature is 50–65 µm) with quite high resolution. However, the inherent material limitations restrict its use in high-performance curvy electronics. To improve the performance of 3D curvy electronics, Sim et al. proposed a new method, which uses conformal additive stamp (CAS) printing [60]. A pneumatically inflated elastomeric balloon is a key component in this method. It is used as the conformal stamping medium, which has a function to form curvilinear 3D shapes. Figure 18 shows the schematic of CAS printing. The high-performance electronics on arbitrary 3D curvy shapes can be created by this method.

Furthermore, researchers have successfully produced curved optoelectronics thanks to the progress in stretchable electronics. To obtain high optical performance, mechanical design plays a significant role in the creation of curved artificial compound eyes. Li et al. examined the mechanics of stretchable MLA using the uniaxial and equibiaxial strains and applied it to microlenses [61]. In this study, a unit cell containing a microlens, supporting the post and base membrane is used to simulate microlens deformation using the finite element analysis technique. The results give valuable insight into building elastomeric mirolens arrays that can withstand an extremely high strain without compromising optical performance. The results and models in this article can provide important guidance for the design of similar systems in future works. Moreover, a recent review has discussed the mechanical design for the artificial compound eye in detail [62].

## 4. Artificial Compound Eye Imaging System Using Metasurface-Based Lens

MLA has gained rapid progress due to the advanced development of micro-optical fabrication. Hence, the MLA is an essential optical device in artificial compound eyes systems because of its outstanding properties, such as its low propagation loss, integration, and miniaturization [63]. Presently, the MLA elements suffer from some disadvantages. For example, the systems are strictly dependent on proper alignment. Most artificial compound eye systems based on planar or curved arrays of microlens are integrated with image sensor arrays. The curved geometry of the artificial compound eye is complicated by limited compatibility with standard microelectronic circuits [50]. As a result, it requires the bulky optical relay systems, the development of complex fabrication and packaging process to produce photodetector arrays [49]. Additionally, MLAs’ inherent chromatic aberration reduces the image quality. This is because the chromatic aberration results in blurring and color distortion. To correct the chromatic aberration, they used several lenses with different focal lengths, achromatic doublet lenses, or apochromatic lenses. Nevertheless, the systems of these lenses are bulky in size, and they limit their application in integrated devices [64]. As an alternative, metasurface can be utilized as an ultrathin optical device to retrieve the chromatic aberration effect. A metasurface lens (another name: Metalens) is a two-dimensional arrangement of subwavelength scatters that manipulates wavefronts, polarization, and light intensity. Instead of relying on gradual phase accumulation, each subwavelength scatter causes an abrupt change in the phase of the incident light. However, an abrupt phase change at an interface, light can be arbitrarily deflected in any direction. Several reviews on the recent developments of metasurfaces have discussed the mechanism of different kinds of metasurfaces in detail [65,66,67]. With the nanostructure of metasurfaces, this kind of artificial compound eye imaging system is a more compact system than traditional types. Moreover, this type can provide a wide FOV and a high-quality image based on the advantages of the metasurface. In this section, we discuss two kinds of artificial compound eye imaging systems using metasurface-based lenses, plasmonic metalens, and dielectric metalens.

### 4.1. Artificial Compound Eye Using Plasmonic Metasurface-Based Lenses

Metalenses utilize metasurface building blocks to exploit the optical characteristics of light. One of the most representative techniques is to create plasmonic effects on the surface [68]. Localized surface plasmons are set up around the surface when light projects into the antenna. An optical resonance of antenna occurs when the antenna lateral size is approximately half the surface plasmonic wavelength. Thus, the incident light is in phase with the coherent oscillation of the free electrons and therefore a current is generated from which light scattering originates. Hence, abrupt phase changes of the scattered light can be induced by varying the antenna’s geometrical parameters. Based on these principles above, Salmanogli et al. illustrated a plasmonic system that can work as a compound eye. The system contains far away plasmonic nanoparticles (NPs) that act independently, like an ommatidium in the compound eye. Using these plasmonic nanoparticles directly induces the entanglement in the system. Thus, the system has a degree of freedom to enhance the point spread function. Moreover, plasmonic nanoparticles can be used as a nanoantenna to radiate the incidence field into the far-field due to their resonance with the plasmonic frequency. This shows that the entangled photons are radiated into the far-field, which will be detected by the system’s detector. Hence, the system can provide an enhanced image resolution [69].

**Figure 19:**
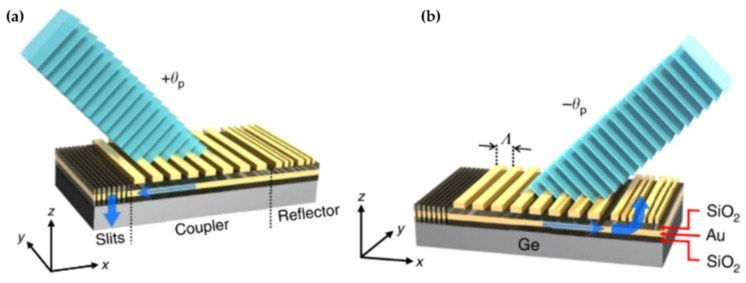
(**a**,**b**) The structure of metasurface. Reproduced from [70]. CC By 4.0.

Furthermore, the artificial compound eye using plasmonic metasurface can provide wider FOV than the traditional one. In fact, Kogos et al. developed artificial apposition compound eyes on planar substrates that can provide an ultrawide FOV greater than 150° in planar lens-less [70]. Figure 19 shows the structure of metalens. In this architecture, each pixel of a standard image-sensor array is coated with an ensemble of metallic plasmonic nanostructures. The ensemble transmits only light incident along a small, geometrically tunable distribution of angles. The optical device consists of simple Ge photoconductors and a composite metasurface, including a metal film stacked with an array of rectangular metallic nanoparticles (NPs). The metasurfaces comprise three sections: A periodic grating coupler, a grating reflector, and a set of slits through underlying metal film. Gold and silicon dioxide (SiO_2_) are used as the choice material for all metallic features and dielectric layers, respectively. The grating coupler is used to control the incident angle of peak detection and propagate the surface plasmon polaritons toward the slits, where they are scattered into the substrate and produce a photocurrent or a grating reflector. By the virtue of its lens-less nature, this approach can provide further miniaturization and higher resolution than previous artificial compound eyes using planar MLA, with a potentially straightforward manufacturing process compatible with the existing image sensor technology. The parameters and performance of these artificial compound eye imaging system are presented in Table 5.

### 4.2. Artificial Compound Eye Using Dielectric Metasurface-Based Lens

**Figure 20:**
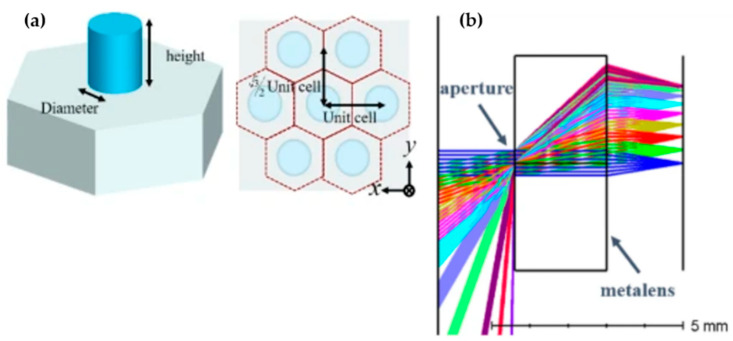
(**a**) The geometry of a unit cell. (**b**) The metalens layout. Reprinted from [71]. CC by 4.0.

Although plasmonic materials can provide strong optical resonances, they have limitations of ohmic loss, where the energy of the incident light is transferred to heat in the material. For applications that require high efficiency of the device, this is non-ideal because the ohmic loss results associated with heat dissipation in plasmonic materials represent a reduction of the overall efficiency. To overcome this limitation, metalens based on dielectric metasurfaces have been studied. Based on the study, metasurface structures from dielectric materials exhibit a lower loss and higher transmission rate than metals’ materials. Typical dielectric materials used include titanium dioxide, silicon, germanium, and tellurium. To derive their benefits from this model, Fan’s group proposed a wide-angle and high-efficiency optical system [71]. They used SIO_2_ and GaN materials for substrate and metalens, respectively (Figure 20). Simulation results of the optical systems showed a wide FOV of over 170° for a wavelength of 532 nm. Furthermore, the focusing efficiencies are as high as 82% at a normal incident and 45% at an incident of 85°. Additionally, the system can correct aberration to provide a more satisfactory image quality.

Even though the chromatic aberration effect of the artificial compound eye system using microlens array reduces the quality of image, the broadband achromatic image is still a big problem for integral imaging. To overcome this issue, another Fan’s group reported a compound eye imaging system consisting of a metalens array that is integrated on a CCD sensor image [64]. The metalens array contains 60 × 60 polarization-insensitive metalenses with nearly diffraction-limited focus using silicon nitride material. The metalens array can be achieved by zero effective material dispersion and different values of the effective refractive index. The metalens array can operate in the visible spectrum from 430 to 780 nm. They demonstrated the image with a high intensity without chromatic aberration. Moreover, the system is suitable for many applications, such as micro-lithography, wavefront sensors, and 3D imaging.

**Figure 21:**
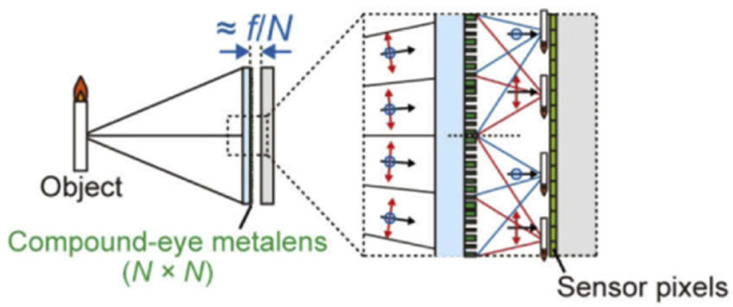
The metalens layout. Reproduced with permission from [72] © The Optical Society.

To overcome the limitations of conventional polarization cameras, another artificial compound eye using dielectric metasurface, which has a low sensitivity and difficulty in device miniaturization is proposed by Miyata et al. The imaging system consists of a typical image sensor and a single metasurface layer for forming a vast number of images while sorting the polarization bases, as depicted in Figure 21. For the metalens, they used silicon nitride nanospots as dielectric building blocks because they are potentially compatible with a CMOS sensor and support metasurface functionalities in visible wavelengths. The metalens was designed for a 100 × 200 µm^2^ aperture, 500-µm focal length, and 520-nm operating wavelength. The metalens are fabricated on a quartz substrate using standard thin-film deposition, single-step electron-beam lithography, and etching processes. The artificial compound eye metalens system has a size of 1 × 1 mm^2^ which created 10 × 10 images with 400 × 400 pixels. This system improves the amount of detected light by a factor of two, while reducing the device thickness to 1/10 of the most prevalent polarization cameras. The parameters and performance of these artificial compound eye imaging system are reported in Table 6.

## 5. Application of Artificial Compound Eye Imaging System

Based on the outstanding advantages of the artificial compound eyes, such as a large FOV, compact thickness, and multi aperture, they have a great potential for various applications. In this section, we briefly introduce some of the artificial compound eye imaging system’s applications.

### 5.1. Imaging with High Resolution, Depth Information, and Multi-Refocusing

**Figure 22:**
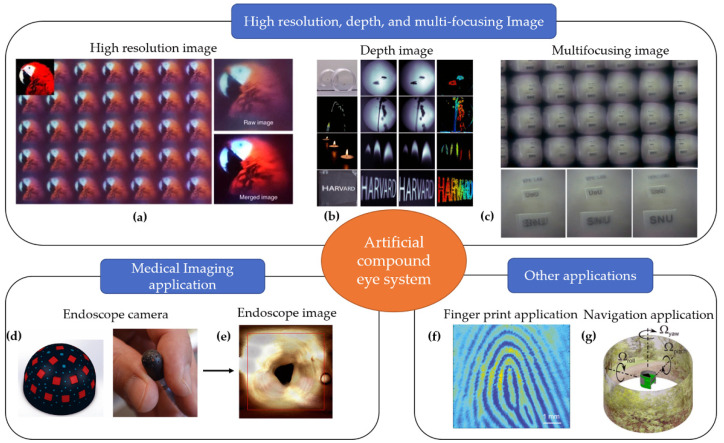
The applications of artificial compound eye systems. (**a**) The high resolution image. Reproduced from [40]. CC by 4.0. (**b**) The depth image. Adapted from [73]. CC by 4.0. (**c**) The multifocusing image. Reprinted from [5]. CC by 4.0. (**d**,**e**) The medical imaging application. Reproduced from [74] with permission from IEEE Publishing. (**f**) The biometric finger print capturing application. Adapted from [75]. CC by 4.0. (**g**) The navigation application. Reprinted with permission from [76] © (2014) Copyright Society of Photo-Optical Instrumentation Engineers.

The artificial compound eye imaging system has lower resolution than the conventional camera because each visual unit has only up to several hundred pixels. However, the partial images of the captured image from the system can be incorporated with each other to form a final image with a high resolution by a reconstruction algorithm. In fact, Tanida et al. used the pixel rearranged method and interpolation method to fuse and retrieve the final image with a high resolution from multiple images with low resolutions [27]. Kim et al. also showed that the integral image reconstructed from array images increases the modulation transfer function (MTF) by 1.57 times compared to a single channel image in an ultrathin camera [40], as shown in Figure 22a.

To obtain depth information of the environment, two cameras or multiple cameras are required to capture the same object in different directions. This is a limitation of the single aperture camera system. It should be noted that an artificial compound eye is a multi-aperture camera that each has an ommatidium in a different view direction. Therefore, the artificial compound eyes can provide 3D information extraction after its one-shot image through digital image processing. Therefore, there are stereo disparities between the individual images that are used to determine the object distance. The multiple base-line method was first used in the TOMBO system to detect object distance and retrieve the 3D image [77,78]. The distances between the three objects from the TOMBO system are 30 mm (dice A), 80 mm (dice B), and 220 mm (Test chart). The extracted distances of the areas of three objects are 31, 104, and 253 mm, respectively. The processing time of the estimation was approximately 3 s. Furthermore, another artificial compound eye system called the COMPU-EYE imaging system for estimating object depths is proposed by Lee et al. [79]. In the COMPU-EYE imaging system, the acceptance angles (45°) are larger than interommatidial angles (1.8°), which cause the overlap between the ommatidial receptive fields. The authors use the disparities between receptive fields and the sparse representation-based classification algorithm to determine object distances and to estimate the object depth. Therefore, the proposed system with this depth estimation method can provide a depth map of the object with 1-mm depth resolution. However, the reconstructed 3D image is not a high-quality image due to the chromatic aberration of the MLA. Fan et al. proposed a silicon nitride metalens array without chromatic aberrations in the visible and use it for integral imaging displays that capture light field information [71]. The 3D view is encoded with algorithms and then a high-quality image with depth information is reconstructed. Recently, inspired by the eyes of the jumping spider, Guo et al. presented a metalens-based depth sensor to perceive depth information (Figure 22b) [73]. The single metalens is responsible in splitting light into two beams and then forming, simultaneously, a pair of different defocused images on the two halves of a single planar photosensor. The authors demonstrated the system that deploys a 3-mm diameter metalens to measure depth over a 10-cm distance range. Compared to other depth sensors, this system is compact, single-shot, and requires a small amount of computation.

Based on the multi aperture structure of the artificial compound eye system, Seo et al. proposed the biomimetic insect compound eye with multiple depth plane images reconstructed based on the taken sub-images with a single shot [5]. Using the 18 partial images of a one single shot, three focused images, which are “SNU”, “UoU”, and “EFE LAB” parting 1 cm, 2 cm, and 3 cm from the imaging system were reconstructed (Figure 22c).

### 5.2. System with a Large FOV for Medical Imaging Application

Recently, the development of artificial compound eye systems has drawn significant attention in diverse applications such as medical endoscopy imaging due to their miniaturized structure, wide FOV, and a high-resolution imaging. Waterproof artificial compound eyes with a variable FOV is presented by Zhou et al. [46]. The FOV of the system has ranged from 0° to 160°. The fabricated MLAs can be used as planar MLAs or curved MLAs as required by various environments, including humid or water. Therefore, the fabricated artificial compound eyes can be incorporated into micro-optical fibers to obtain a wide FOV for actual transmission imaging in medical endoscopy.

Cogal et al. also reported a miniaturized high definition vision system inspired by insect eyes that can work in dark environments for endoscopy application [74]. The system consists of 24 single cameras mounted on a hemispherical surface with a 5-mm radius, as described in Figure 22d,e. Unlike the artificial compound eye with a similar size, the system features 1000× a more spatial resolution. The system has a 180° × 180° field of view while providing more than 1.1 megapixels. Moreover, it can generate a 25-fps video with a 1020 × 1080-pixel resolution at a 120-MHz processing clock frequency.

In magnetic resonance imaging (MRI) application, a highly compact (6-cm detector ring size) MRI SPECT system called MRC-SPECT-II based on artificial compound eye camera is presented by Lai et al. [80]. The system has 24 detector rings and 1536 micro pinhole camera elements. The FOV is up to 360° and has a 0.65-mm temporal resolution. The peak sensitivity of the system could reach 1.5% in the central FOV and decreases at the position away from the center FOV.

### 5.3. Other Applications

A compact compound eye imaging module based on the diffractive MLA for biometric fingerprinting capturing is proposed [81]. The system composed of a top glass plate, a diffractive MLA, and an image sensor. The top glass plate has two roles, one serves as the touch panel for fingerprint collection, and another one serves as backlighting waveguide for illumination. The fingerprint image captured by the 5 × 4 diffractive MLA, includes 20 unit-images. The complete fingerprint image is generated from 20 sub-images by an imaging reconstruction method. Ting et al. introduced another artificial compound eye imaging system for fingerprint imaging [75]. The system included an a-Si metalens working 940 nm, fabricated on 12-inch glass wafer using CMOS sensor technology. The number aperture and the focal spot size of the metalens are measured to be 0.496 and 1.26 µm, respectively. To evaluate this system, the fingerprint textures image captured by moving the metalens (3 × 3 image array). The results showed that the image of fingerprint textures is a high-quality image (Figure 22f). In addition, using the proposed metalens-array makes the system more compact.

For navigation purposes, the CurvACE system was reported to have been directly applied in the autonomous navigation system [76,82], as shown in Figure 22g. The optical method was used for navigation in the two systems. The CurvACE consists of a curved matrix of a 42 × 15 artificial ommatidia and has a FOV of 180°. The CurvACE is placed inside a circular arena lined with a natural print picture for navigation. For the Lucas–Kanade method, a 2D optic flow is calculated at about 1000 optic flow vectors per second across a FOV 180° × 60°. For the I2A method, a maximum processing rate of about 20,000 optical flow vectors per second is demonstrated.

## 6. Conclusions and Outlook

We reviewed and analyzed the system design and applications of two types of artificial compound eye using MLA and metasurface-based lens. Despite researchers having tried various techniques to create artificial compound eye systems for many applications, there are still many areas to be improved and explored. We list some problems that need to be addressed in future works for these types of artificial compound eye systems. In an artificial compound eye that uses an MLA, the planar type is a simple system that can easily be fabricated but the major drawback of this structure is the narrow FOV. As a result, increasing the FOV is a significant problem that needs to be solved to improve the imaging performance of this sort of artificial compound eye imaging system. The curved type had a large FOV and a high resolution. Nevertheless, to create a curved artificial compound eye, image sensors are still manufactured on a flat substrate, resulting in a high level of fabrication complexity for correcting distortion. Therefore, understanding how to connect curved microlens to the planar image sensor is an issue that must be addressed. In addition, more modern manufacturing techniques should be studied for the curved image sensor in future works. For artificial compound eyes that use metasurface, even though, we improved the disadvantage of the microlens-array lens, however its resolution is still not on the level of currently commercialized ones. Hence, how to treat the trade-off between miniature device and resolution is a challenging issue in enhancing the imaging performance of artificial compound eyes.

In conclusion, the imaging performance of the artificial compound eye imaging system must be considerably improved in comparison with commercial cameras and natural compound eye for a wider range of applications.

## Figures and Tables

**Table 1:** A comparison of the single-aperture, the apposition, and the superposition eye [24,25].

Parameter	Human	Bee	Moth	Shrimp Acetes Sibogae	Bibio Marci
Structure	Single aperture	Apposition compound	Refractive Superposition compound	Reflective Superposition compound	Neural superposition compound
Light habitat	Diurnal	Diurnal	Nocturnal	Nocturnal	Diurnal
Lens diameter (mm)	7	0.025	0.4	0.7	0.021
Focal length (mm)	23	0.06	0.17	0.3	0.07
Receptor diameter (um)	2	1.5	8	-	-
F-number (F/#)	3.3	2.4	0.4	0.4	0.6
Sensitivity (${\mu m}^{2}=10^{-12}W/\left(W/m^{2}\right)$)	0.23	0.24	218	0.327	0.2742–1.2611
Acceptance angle (°)	0.007	1.9	<13	5.6	2.0
Interreceptor angle d/f (°)	0.005	-	3	-	2.8
Interommatidial angle (°)	-	0.95	-	2.8	1.6
Resolution (LP/°)	100	0.52	>0.08	-	-

(-) means unknown.

**Table 2:** The parameters and performance of the planar-type artificial compound eye system.

Author	System Name	Optic Component Integration	Focal Length	FOV	Resolution (Pixels)	Imaging Performance
Tanida et al., 2001 [26]	TOMBO	CCD camera	650 µm	-	320 × 240	Low resolution image.
Tanida et al., 2003 [28]	TOMBO	CMOS image sensor	1.3 µm	-	360 × 360	Low resolution image with pixel number 180 × 180.
Duparre et al., 2004 [30]	APCO	CCD image sensor	300 µm	21°	-	Ghost image due to crosstalk between channels.
Duparre et al., 2005 [32]	Cley	CCD camera	2.75 µm	70° × 10°	700 × 500	Low contrast image, a resolution of 3.3 LP/°.
Duparre et al., 2005 [34]	Chirped APCO	CMOS image sensor	145 µm	64.3°	60 × 60	Low resolution image.
Bruckner et al., 2007 [33]	Artificial neuro superposition eye	CMOS image sensor	-	23° × 21°	70 × 53	Low resolution image. MTF is 0.5 when spatial frequency is 0.4.
Stollberg et al., 2009 [23]	Gabor Superlens	CCD image sensor	2.4 mm	29°	156 × 156	Low resolution image, maximum resolution in image space is 49 LP/mm.
Bruckner et al., 2010 [36]	Thin wafer level camera lenses	CCD image sensor	-	58° × 46°	700 × 550	Low resolution image, distortion image.
Meyer et al., 2011 [39]	oCLEY	CMOS image sensor	1.39 mm	53.2° × 39.9°	640 × 480	Spatial frequency has maximum value of 222 cycles/mm, a high angular resolution, to reach the VGA resolution.
Seo et al., 2018 [5]	Microlens integrate gold layer screening camera	CCD image sensor	1.442 mm	100°	1920 × 1080	High resolution image.
Kim et al., 2020 [40]	Ultrathin arrayed camera	CMOS image sensor	150 µm	73°	8 Megapixel	High resolution image, MTF50 obtains 202 cycles.mm^−1^.

(-) means unknown; CCD: Charge-coupled device; CMOS: Complementary metal oxide semiconductor; MTF: Modulation transfer function.

**Table 3:** The parameters and performance of the artificial compound eye with curved MLA integrated to a planar image sensor.

Author	Optical Components Integration	Focal Length	Numerical Aperture (NA)	FOV	Imaging Performance
Liu et al., 2012 [41]	Laser confocal microscope	92.4 µm	0.3	162°	No distortion image.
Bian et al., 2016 [43]	Microscope and CCD camera	606.69 µm	0.08	-	Clear and sharp image.
Huang et al., 2017 [44]	Microscope system	23.72 µm	0.4	-	Little distortion image.
Zhou et al., 2020 [46]	CCD imaging sensor	18.98 µm	0.46	161°	No distortion image, the FWHM intensity 3.2 ± 0.2 µm and 3.0 ± 0.1 µm along the x and y direction, respectively.
Deng et al., 2016 [47]	CCD camera	32.8 µm	0.5	140°	Distortion free image, the FWHM intensities are 1.7± 0.1 µm (x axis) and 1.6 ± 0.3 µm (y axis).
Li et al., 2020 [48]	Microscopy system	100 µm	0.4	140°	Distortion image, the FWHM intensity is 3 µm for two directions.
Wang et al., 2019 [49]	CMOS imaging sensor	2.09 mm	0.125	180°	Good imaging performance, no distortion image.
Keum et al., 2018 [51]	Microscope system	1.4 mm	-	68°	Image with high resolution, MTF50 was 154 cycle.mm^−1^.
Lian et al., 2020 [52]	CMOS imaging sensor	1.2 mm	-	109°	Distortion image.

(-) means unknown; CCD: Charge-coupled device; CMOS: Complementary metal oxide semiconductor; MTF: Modulation transfer function; FHWM: Full width at half maximum.

**Table 4:** The parameters and performance of the artificial compound eye imaging system with curved microlens array integrated to a curved image sensor.

Author	System	Optic Component Integration	FOV	Acceptance Angle	Interommaditium Angle	Imaging Performance
Song et al., 2013 [55]	Arthopod inspired camera	CMOS image sensor	160°	4.2°	4.2°	High resolution image without aberration.
Floreano et al., 2013 [56]	CurveACE	CMOS image sensor	180° × 60°	9.7°	11°	High resolution image, motion detection capabilities, crosstalk prevention.

CMOS: Complementary metal oxide semiconductor.

**Table 5:** The parameters and the performance of the artificial compound eye using plasmonic metasurface-based lens.

Author	Size	FOV	Operating Wavelength	Focal Length	Imaging Performance
Salmanogli et al., 2018 [69]	Plasmonic nanoparticle with a radius around 50–100 nm	-	808 nm	68 µm	High image resolution.
Kogos et al., 2020 [70]	60-nm thickness of SiO_2_ layer, 100 nm thick gold film with a 5-nm Titanium	150°	1550 nm	10 mm	High-quality images with a 5280-pixel array at signal noise ratio of 73 dB.

(-) means unknown.

**Table 6:** The parameter and performance of the artificial compound eye using dielectric metasurface-based lens.

Author	Size	FOV	Operating Wavelength	Numerical Aperture	Focal Length	Imaging Performance
Fan et al., 2020 [71]	Diameter of metalens is 110 µm	170°	532 nm	0.64	2 mm	Focusing efficiency are 82% at a normal incident and 45% at an incident of 85°. High image quality without aberrations.
Fan et al., 2019 [64]	Thickness is 400 nm, metalens with a diameter of 14 µm	-	430 to 780 nm	0.086	81.5 µm	Focusing efficiency can be obtained in the range of 36–55% at all visible wavelengths.
Miyata et al., 2020 [72]	Size of metalenses is 100 × 200 µm^2^	-	520 nm	0.8	500 µm	Image size is 400 × 400 pixels. Image is clear at 520 nm wavelength.

(-) means unknown.

## Data Availability

Not applicable.
